# The prevalence of overweight and obesity among Iranian military personnel: a systematic review and meta-analysis

**DOI:** 10.1186/s12889-019-6484-z

**Published:** 2019-02-06

**Authors:** Yahya Salimi, Maryam Taghdir, Mojtaba Sepandi, Ali-Akbar Karimi Zarchi

**Affiliations:** 1 Health Research Center, Life Style Institute, Baqiyatallah University of Medical Sciences, Tehran, Iran; 2 Department of Nutrition and Food Hygiene, Faculty of Health, Baqiyatallah University of Medical Sciences, Tehran, Iran; 3 Department of Epidemiology and Biostatistics, Faculty of Health, Baqiyatallah University of Medical Sciences, Tehran, Iran

**Keywords:** Prevalence, Overweight, Obesity, Systematic review, Meta-analysis, Military personnel

## Abstract

**Background:**

The overweight and obesity among military personnel, as an occupational group, beside the health issues, might affect their military performance. A systematic review and meta-analysis were conducted to estimate the pool prevalence of overweight and obesity among Iranian military personnel.

**Methods:**

The national databases including Science Information Database, MagIran, and the following international databases; Web of Science, Medline via PubMed, and Scopus were searched, up to December 2017, for relevant published studies without time limitation.

**Results:**

Totally,1431 studies were reterived and 10 studies included in meta-analysis. The pooled prevalence of overweight and obesity were 41% (95%CI:26, 57%) and 13% (95%CI:10, 17%), respectively. In the subgroup analyses, a lower and higher prevalence of overweight was reported in the ground (12%) and Navy (69%) forces military, recpectively. For the obesity, the air forces had the lowest prevalence (11%) and the ground and Navy forces military had highest prevalence (15%). The prevalence of overweight and obesity were slightly higher in studies conducted after 2014.

**Conclusion:**

Our findings suggest a high prevalence of overweight and obesity in the military personnel as a high-risk occupational group. Owing to the high observed heterogeneity among the included studies, large representative studies are needed to estimate the prevalence of overweight and obesity in the military personnel.

## Background

Global data indicate that overweight and obesity are two of the major public health concerns in all population age groups [1]. According to the World Health Organization (WHO) report, during the past four decades, prevalence of overweight and obesity has been dramaticaly increased in the general population [1]. In 2016, 39 and 13% of adults in the world were overweight and obese, respectively [1]. The overweight and obesity are defined as abnormal or excessive fat accumulation in the body [1, 2]. Based on the WHO classification, individuals with a body mass index (BMI) between 25 and 30 and more than 30 are considered as overweight and obese, respectively [2]. Obesity is caused by several factors including genetics, metabolic, socioeconomic status, enviromental and behavioral factors [3].

The underlying reported association between overweight, obesity and many chronic diseases such as cardiovascular disease, diabetes, rheumatoid arthritis, hypertension, physical inability and respiratory illness have reoinforced the importance of these public health concerns [1, 4, 5]. Several studies have shown that obese or overweight people are more likely to experience a higher morbidity and mortality than those with normal weight [6–9]. It is now well-acknowledged that occupation-related factors could have an important role in the prevalence of overweight and obesity [10–13].

Military personnel as an occupational group are at higher risk of stressful conditions, exposure to death or harmful agents as well as imposed restriction on food selection or availability. Having an ideal body weight and fitness is a fundamental principle in military forces recruitment. Beside of an imposed undesirable health outcome, overweight and obesity can affect the productivity of military forces during both peacetime and combating operations [14]. Considering the physical training of military forces, it is expected that their weight would be normal than the general population. Nonetheless some evidence suggest that the overweight and obesity prevalence have an increasing trend in the US military over the past two decades. [15, 16] Several studies in different countries reported a relatively high prevalence of overweight and obesity among military forces [17–21]. Fajfrová et al., in their 11-years follow-up study among the professional soldiers in Czech Army, reported a gradually increased prevalence of overweight (52 to 57.1%) from 1999 to 2009 [19]. In Saudi Arabia, prevalence of overweight and obesity among active military personnel were estimated at 40.9 and 29%, respectively [18]. Another study in Greek warship personnel reported prevalence of overweight and obesity as 26.5 and 4.7%, respectively [20].

In Iran, a various prevalence of overweight and obesity has been reported among military forces, ranged from 11.5 to 69% and 6.2 to 23.1%, respectivly [22–31].

Notwithstanding the diversity in the reported prevalence of overweight and obesity, a pooled estimation of overweight and obesity prevalence is greatly warranted for planinng effective therapeutic and preventive interventions among military forces.

We aimed in this study to estimate a pooled prevalence of overweight and obesity among military personnel in Iran by using a systematic review and meta-analysis.

## Methods

Preferred Reporting Items for Systematic Reviews and Meta-Analyses (PRISMA) checklist and flow diagram were used for designing and reporting the procedure [32].

### Search strategy

The following national databases including Science Information Database (up to December 2017); Magiran (up to December 2017), as well as the following international databases including Web of Science (up to December 2017); Medline via PubMed (up to December 2017); Scopus (up to December 2017) were searched. The search strategy for PubMed was as follow: ((“Body Mass Index” OR “Obesity” OR “Obesity, Abdominal” OR “Overweight” [Mesh]) AND (“Military Personnel” OR “Army personnel” OR “armed forces” OR “security forces” [Mesh]) AND Iran). Similar specification was used for the other databases. Also, a manual search of references list in selected articles was conducted. In the case of unavailability of full texts or missed information, we attempted to obtain the full text or information from authors by email.

### Eligibility criteria

We included all cross-sectional studies that have examined the prevalence of obesity and overweight using BMI in Iranian military forces, regardless of time and location of the studies, age and military service type of the studied population, and publication language (Persian or English) of the studies. In the present review, the outcome was the prevalence of obesity and overweight in the Iranian military. Since BMI has been recommended as a standard and common measure for overweight and obesity [1], we excluded the studies that reported the prevalence of overweight and obesity based on waist circumference or hip-waist ratio.

### Selection of studies

For appropriate selection of the realted articles, two reviewers conducted literature search independently. The two reviewers were not blinded to the articles’ author names, journal name and results. Any discrepancy between the two reviewers, it was resolved by negotiation or with the guidance of a third person.

### Data extraction and methodological quality assessment

After selecting the studies, the related variables in each study including the study type, sample size, number of overweight and obesity patients, demographic characteristics of participants, time and place of study were entered in pre-designed Microsoft excel data sheet. Strengthening the Reporting of Observational studies in Epidemiology (STROBE) statement [16] was used for evaluating potential biases in the included studies. Six methodological related items from STROBE were applied on the selected studies, including: 1) “description of study design”; 2) “specific objectives or hypothesis”; 3) “description of setting, locations, and time scales for outcomes”; 4) “eligibility criteria, major sources and methods of recruiting participants”; 5) “clearly define primary outcome”; and 6) “description of how a study sample was arrived at”. Each item was scored as fully satisfied (2 score point), partially satisfied or can’t tell (1 score point) and unsatisfied (0 score point). Finally, the studies with a total score of ≥10,7 to 9 and ≤ 6 classified as high-quality, intermediate-quality, and low-quality studies, respectively. The quality assesment of the studies was carried out independently by two reviewers (YS and MT). Any disagreement between the two reviwers was resolved through negotiation with the third reviewer (MS). The exclusion of studies was not based on the quality assessment scores.

### Statistical analysis

We examined statistical heterogeneity using the chi-square χ2 test. *P*-value less than 0.05 was considered as heterogeneity. Inconsistency between the studies was evaluated using the I^2^ statistic, higher I^2^ values indicating greater variability among studies than would be expected by chance alone (range: 0–100%) [33]. We also estimated the between-study variance using tau-square (Tau^2^) statistic [34]. The Begg and Egger tests were conducted to assess the publication bias [35, 36].

Meta-analysis to estimate pooled prevalence was conducted using a random-effects model [37] with 95% confidence interval (CI). We conducted four subgroup analysesto examine the possible effects of the included studies quality, geographic region of Iran, year of study, and type of military force.

The Stata version 14.0 software (Stata Corp, College Station, TX) was used for the data analysis and all statistical tests were two-tailed. For Begg and Egger tests, *p*-value less than 0.1 was considered as a statistically significant, but for other tests p-value less than 0.05 was considered as a statistically significant.

## Results

### Study characteristics

**Fig. 1:**
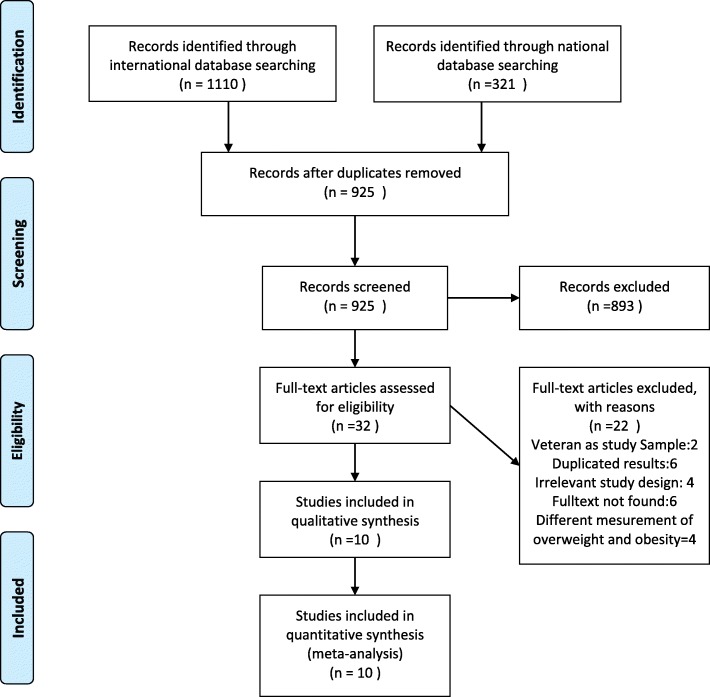
Flow diagram for the study selection

Totally 1431 records up to December 2017 were retrieved using our search strategy. Of them 506 duplicate records were excluded. We also excluded 893 articles after screening their title and abstract. The full text of the remaining 32 studies were screened, and 22 studies were excluded (Fig. 1). Finally, 10 full-text articles were included in the meta-analysis that estimated the overweight and obesity pooled prevalence. Overally, these 10 studies included 17,920 participants. Two of the included studies did not report the obesity information, therefore the summary measure for the obesity outcome was estimated for 8 studies. The Fig. 1 depicts a diagram regardingthe flow of information in this review study.

The included studies were conducted mainly Tehran (*n* = 6). One study was conducted at the national level. Five of the 10 studies included a sample of all military force units and one study recruited a sample of navy force unit. Table 1 shows the characteristics of the 10 included studies in the meta-analysis.

**Table 1:** Characteristic of included studies in meta-analysis

First author	Year of pulication	Mean age (SD) or range	Sample size	Location (city/province)	Type of military force	Prevalence of overweight	Prevalece of obesity	Quality of study
Fallahi [22]	2013	39.4 (7.70)	100	Center of Iran (Tehran)	All forces	0.484	0.108	Intermediate
Karimi Zarchi [31]	2010	30.4 (8.80)	405	Center of Iran (Tehran)	All forces	0.209	–	Low
Pourtaghi [30]	2014	18–30	12,635	Center of Iran (Tehran)	Ground force	0.115	–	Low
Iravani [25]	2010	35 (7.50)	380	South of Iran	Ground force	0.506	0.146	High
Khoshdel [26]	2012	37.4 (6.40)	96	South of Iran	Air force (Parachutists)	0.469	0.105	Intermediate
Payab [29]	2017	33.37 (7.75)	2200	Center of Iran (Tehran)	All forces	0.476	0.151	Intermediate
Ghanbary sartang [23]	2016	36.5 (3.14)	100	Center of Iran (Tehran)	Navy	0.690	0.150	High
Maleki [27]	2016	20–60	1000	Center of Iran (Tehran)	Air force	0.417	0.110	Low
Marzabadi E [28]	2011	25–50	749	National	All forces	0.350	0.062	High
Angoorani [24]	2014	37 (7.0)	255	Center of Iran (Tehran)	All forces	0.490	0.231	High

### Estimated prevalence and heterogeneity of studies

**Fig. 2:**
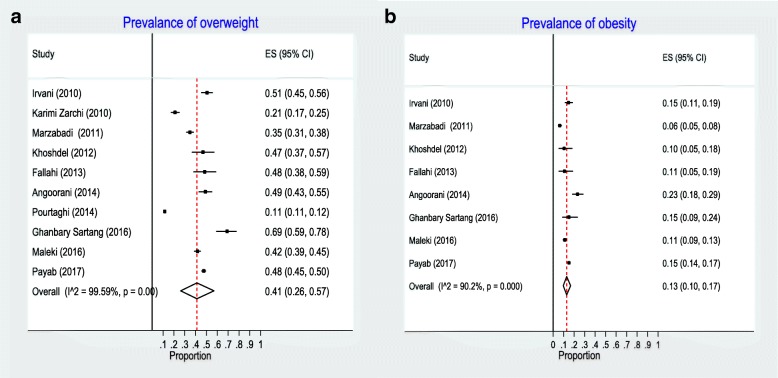
Point prevalence of overweight (**a**) and obesity (**b**) in Iranian millitary population

Based on the random-effects model, the pooled prevalence of overweight was 0.41 (95% CI:0.26, 0.57), with a considerable heterogeneity (chi^2^ (9) = 2210.48, *p* < 0.001; I^2^ = 99.59%). Also, the pooled prevalence of obesity was 0.13 (95% CI:0.10, 0.17), with a remarkable heterogeneity (chi^2^ (9) = 71.24, p < 0.001; I^2^ = 90.17%) (Fig. 2a and b). The between-study variances tau^2^ for the overweight and obesity were 0.26, and 0.02, respectively. The highest prevalence of overweight (69%) was observed in the study by Sartang et al. [23], and the lowest prevalence of overweight (11.5%) was reported by Pourtaghi et al. [30].

The results of Begg test for overweight was statistically significant (*p* = 0.005), whereas the Egger test for overweight was not statistically significant (*p* = 0.421). For obesity, the results of Egger and Begg test indicating that there is no publication bias (*p* = 0.588 and *p* = 0.805, respectively).

### Quality assessment

All of the included studies specified their objectives or hypothesis. Only one study did not report the type of the study design [30]. Two of the studies did not provide clear description of the study setting/location and time scale for the outcomes [30, 31]. Also, only one study reported that how the study sample size was arrived [25]. Two of the included studies did not report the obesity information [30, 31]. The quality of the included studies have been presented in Table 1.

### Subgroup analysis

We conducted four subgroup analysis to address the effect of the location of the study samples, year of publication, type of military force, and quality of the included studies as potential source of the observed heterogeneity. Although, the heterogeneity was still appreciable for all subgroups. In the subgroup analysis, a lower prevalence of overweight was reported in the ground forces military. For the obesity, the air forces had the lowest prevalence. The prevalence of both overweight and obesity were slighty higher in the recent studies (After 2014 vs. before 2014) (Table 2).

**Table 2:** Subgroup analysis of overweight and obesity prevalence based on location of the study samples, year of studies publication, type of military force, and quality of the included studies

	Overweight		*p*-value*	Obesity		*p*-value*
	Pooled prevalence	95% CI		Pooled prevalence	95% CI	
Location of the study samples						
Center of Iran (Tehran)	0.35	0.17, 0.56	*p* < 0.001	0.15	0.11, 0.19	*p* < 0.001
South of Iran	0.55	0.43, 0.67	*p* < 0.001	0.14	0.11, 0.17	*p* < 0.001
National	0.35	0.31, 0.38	*p* < 0.001	0.06	0.05, 0.08	*p* < 0.001
Year of the studies publication						
Before 2104	0.40	0.28, 0.52	*p* < 0.001	0.10	0.06, 0.16	*p* < 0.001
After 2014	0.43	0.20, 0.67	*p* < 0.001	0.15	0.11, 0.20	*p* < 0.001
Type of military force						
All forces	0.40	0.29, 0.51	*p* < 0.001	0.13	0.07, 0.21	*p* < 0.001
Ground force	0.12	0.12, 0.13	*p* < 0.001	0.15	0.11, 0.19	*p* < 0.001
Aire forces	0.42	0.39, 0.45	*p* < 0.001	0.11	0.09, 0.13	*p* < 0.001
Navy	0.69	0.59, 0.78	*p* < 0.001	0.15	0.09, 0.24	*p* < 0.001
Quality of the included studies						
High	0.50	0.38, 0.63	*p* < 0.001	0.14	0.07, 0.23	p < 0.001
Intermediate	0.48	0.46, 0.50	*p* < 0.001	0.14	0.11, 0.17	*p* < 0.001
Low	0.24	0.07, 0.47	*p* < 0.001	0.11	0.09, 0.13	*p* < 0.001

*p-value for Chi^2^ statistic for heterogeneity

The prevalence of overweight in the high quality studies (0.50; 95% CI: 0.36, 0.63) was higher than intermediate (0.48; 95% CI: 0.46, 0.50) and low quality (0.24; 95% CI:0.07, 0.47) studies.

## Discussion

The aim of the present review study was to estimate the pooled prevalence of overweight and obesity among Iranian military personnel. Our findings showed that the pooled prevalence of overweight and obesity were high (41 and 13%, respectively). However, the estimated prevalence in our study was lower than those reported among 40-year-old Royal Brunei armed forces (60.1 and 12.1%, respectively) [38].

Our estimated prevalence of overweight is comparable with those reported by Horaib et al. conducted among 10,500 active military personnel in Saudi Arabia (41% VS. 40.9%) [18]. However, the prevalence of obesity in our study was much lower than the Horaib et al’ study (13% VS. 29%). One study among 30–39 years old Nigerian military population found that 40.4% of personnel were overweight or obese [39]. Several studies indicated an increasing trend in the prevalence of overweight and obesity in U.S. army [15, 40, 41]. In a recent paper by Hurby et.al, in which a trend in overweight and obesity from 1989 to 2012 among U.S. army were explored, the prevalence of overweight increased from 25.8 to 37.2% and obesity increased from 5.6 to 8.0% [41]. Another study that was conducted among 18-year-old applicants in the U.S. military from 1993 to 2006 reported that the prevalence of overweight increased from 22.8% in 1993 to 27.1% in 2006, and the prevalence of obesity increased from 2.8% in 1993 to 6.8% in 2006 [40]. Moreover, in other countries an increasing trend in the prevalence of overweight and obesity have also been reported. For example, Fajfrová et al., during a 11-year follow-up period among professional soldiers in Czech Army, demonstrated that prevalence of overweight gradually increased from 52 to 57.1% in 1999 to 2009 [19]. Another study in Greek warship personnel reported prevalence of overweight and obesity as 26.5 and 4.7%, respectively [20]. The worldwide prevalence of overweight and obesity in general population has been increased [42]. This increase can affect military personnel as a subgroup of general population. However, in our review only four high quality studies were included that might affect the estimated pooled prevalence.

A body of literature shows that overweight and obesity can play an important role in developming of cardiovascular disease, diabetes, rheumatoid arthritis, hypertension, physical inability, respiratory illness and risk of injuries [1, 4, 5, 43]. In a study by Rappole et.al among USA’s male army soldiers in an operational brigade, it is demonstrated that higher BMI was associated with risk of injury [43]. In addition, psychological distress could be increased by overweight and obesity [44, 45]. These physical and psychological disorders can affect the best performance of military personnel.

According to the related literature, there may be number of possible explanations for the high observed prevalence of overweight and obesity among military personnel; first, military personnel are at higher risk of stressful conditions, exposure to death or harmful agents and ongoing sleep deprivation. Current evidence shows that occupation-related factors have high impacts on the prevalence of overweight and obesity [10–13]. Several researches reported that overweight and obesity can be associated with work environment and higher work related-stress [10–12, 14], including poor eating habits, intake higher energy, saturated fat, and sugar [46, 47]. Stressful environment may result in increasing appetite through cortisol reactivity [48, 49]. However, some people may lose weight when affected by stress [50]. In addition, it is well known that sleep disorder is associated with body weight and adiposity [51, 52].

Second, food selection or availability may have an important role in overweight and obesity among military personnel [53, 54]. It is important to encourage general population to healthier foods consumption as opposed to energy-dense and low nutrient foods in the childhood [54]. Also, it is recommended that a healthier food menu along with food portion control should be provided at military dining facilities.

Third, BMI as one of the inclusion criteria in the present study is the mostly common population-level index for measuring overweight and obesity. Military personnel are often assumed to be younger and more active people than general population. It should be noted that BMI may not accurately reflect the body fat and its distribution [55, 56]. One study that was conducted to evaluate the validity of different measures in categorizing overweight or obesity among overweight or obese active duty military personnel in USA, found a high rates of false negatives for both waist circumference and BMI compared to body fat percentage [57]. Therefore, BMI cannot differentiate between the adipose tissue and muscle mass in military personnel [58].

According to our results, there was a considerable heterogeneity among the included studies that explored with the subgroup analyses. The subgroup analyses indicated that the prevalence of overweight among Ground (12%) and Navy forces (64%) were lowest and highest, respectively. The studies that were conducted recently (after 2014) reported a slightly higher prevalence for both overweight and obesity. The prevalence of both overweight and obesity in high-quality studies were higher than others groups. The low-quality studies reported a lower prevalence of overweight and obesity compared to the intermediate-quality studies. Previous evidence shows that small studies, due to lower methodological quality and larger heterogeneity, tend to report not only larger effect sizes, but also less precise estimates of between-study heterogeneity [59, 60].

Also, we did not include unpublished studies which might be smaller studies that were not submitted or accepted for publication. This limitation may affect the estimated pooled prevalence in our study. The findings of the included studies should be interpreted with caution owing to the inconsistency in sampling designs, sample sizes, and response rates. The present review suggests that further studies should to pay more attention to basic methodological aspects to estimate valid and precise prevalence of overweight and obesity in military personnel.

This study had four main limitations. First, unpublished researches were not included. It’s may be one of the possible explanations for significant result of Egger test for overweight. Although, the Begg test have very low power to detect publication biases, hence shows a non-significant result for overweight. Second, six of the included studies had not high quality, which may affect the pooled prevalence. Third, for obesity, two studies could not be included because they did not report the obesity prevalence. Finally, we were not able to reduce the appreciable observed heterogeneity by using sub-group analysis.

## Conclusion

The present systematic review and meta-analysis highlighted that the overall prevalence of overweight and obesity among military personnel is considerable.

Considering the high observed heterogeneity between the included studies large representative studies are needed to estimate the prevalence of overweight and obesity in the military personnel. It may be useful to consider selective prevention programs that address overweight and obesity based on type of military force.

## Abbreviations

BMI: Body mass index
CI: Confidence interval
PRISMA: Preferred Reporting Items for Systematic Reviews and Meta-Analyses
STROBE: Strengthening the Reporting of Observational studies in Epidemiology
WHO: World Health Organization

## References

[1] WHO: Obesity and Overweight factsheet from the WHO. Retrieved December 13, 2018, from https://www.who.int/news-room/fact-sheets/detail/obesity-and-overweight

[2] WHO: Physical status: the use and interpretation of anthropometry: report of a WHO expert committee. Geneva; 1995 *WHO technical report series* 2014, 854. 8594834

[3] Campfield LA, Smith FJ (1999). The pathogenesis of obesity. Best Pract Res Clin Endocrinol Metab.

[4] Zimmermann-Belsing T, Feldt-Rasmussen U (2004). Obesity: the new worldwide epidemic threat to general health and our complete lack of effective treatment. Endocrinology.

[5] Reilly JJ, Kelly J (2011). Long-term impact of overweight and obesity in childhood and adolescence on morbidity and premature mortality in adulthood: systematic review. Int J Obes.

[6] Adams KF, Schatzkin A, Harris TB, Kipnis V, Mouw T, Ballard-Barbash R, Hollenbeck A, Leitzmann MF (2006). Overweight, obesity, and mortality in a large prospective cohort of persons 50 to 71 years old. N Engl J Med.

[7] Fontaine KR, Redden DT, Wang C, Westfall AO, Allison DB (2003). Years of life lost due to obesity. Jama.

[8] Reilly JJ, Methven E, McDowell ZC, Hacking B, Alexander D, Stewart L, Kelnar CJ (2003). Health consequences of obesity. Arch Dis Child.

[9] Solomon CG, Manson JE (1997). Obesity and mortality: a review of the epidemiologic data. Am J Clin Nutr.

[10] Schulte P, Wagner G, Downes A, Miller D (2008). A framework for the concurrent consideration of occupational hazards and obesity. Ann Occup Hyg.

[11] Luckhaupt SE, Cohen MA, Li J, Calvert GM (2014). Prevalence of obesity among US workers and associations with occupational factors. Am J Prev Med.

[12] Gu JK, Charles LE, Bang KM, Ma CC, Andrew ME, Violanti JM, Burchfiel CM (2014). Prevalence of obesity by occupation among US Workers: the National Health Interview Survey 2004–2011. J Occup Environ Med.

[13] Allman-Farinelli MA, Chey T, Merom D, Bauman AE: Occupational risk of overweight and obesity: an analysis of the Australian health survey. J Occup Med Toxicol (London, England) 2010, 5:14–14. 10.1186/1745-6673-5-14 PMC2894850 20550716

[14] Bernaards CM, Proper KI, Hildebrandt VH (2007). Physical activity, cardiorespiratory fitness, and body mass index in relationship to work productivity and sickness absence in computer workers with preexisting neck and upper limb symptoms. J Occup Environ Med.

[15] Lindquist CH, Bray RM (2001). Trends in overweight and physical activity among US military personnel, 1995–1998. Prev Med.

[16] Reyes-Guzman CM, Bray RM, Forman-Hoffman VL, Williams J (2015). Overweight and obesity trends among active duty military personnel: a 13-year perspective. Am J Prev Med.

[17] Aliyu S, Oyeyemi A, Udoh D (2014). Prevalence of overweight/obesity and undiagnosed hypertension among military personnel in Maiduguri, Nigeria. J Nov Physiother.

[18] Horaib GB, Al-Khashan HI, Mishriky AM, Selim MA, AlNowaiser N, BinSaeed AA, Alawad AD, Al-Asmari AK, AlQumaizi K (2013). Prevalence of obesity among military personnel in Saudi Arabia and associated risk factors. Saudi Med J.

[19] Fajfrová J, Pavlík V, Psutka J, Husarová M, Krutišová P, Fajfr M (2016). Prevalence of overweight and obesity in professional soldiers of the Czech Army over an 11-year period. Vojnosanit Pregl.

[20] Mazokopakis EE, Papadakis JA, Papadomanolaki MG, Vrentzos GE, Ganotakis ES, Lionis CD (2004). Overweight and obesity in Greek warship personnel: prevalence and correlations. The European J Public Health.

[21] Ray S, Kulkarni B, Sreenivas A (2011). Prevalence of prehypertension in young military adults & its association with overweight & dyslipidaemia. Indian J Med Res.

[22] Fallahi A, Fakhroddin F (2013). A R: survey on body mass index and eating habits as chief variables of lifestyle in active duty military personnel in 2011-2012. Iran J Police Med.

[23] Ghanbary Sartang A, Ashnagar M, Habibi E, Nowrouzi I: The relationship of body mass index and waist-hip ratio with shift work among military personnel in 2016.

[24] Hooman Angoorani M, Naghavi-Moghadam AA, Khoshdel AR. Body mass index and composition in physical preparedness of Iranian military personnel. Annals Military Health Sci Res. 2014:70.

[25] Iravani S, Sabayan B, Sedaghat S, Heydari S, Javad P, Lankarani K, Khoshdel A (2010). The association of elevated serum alanine aminotransferase with metabolic syndrome in a military population in southern Iran. Age (Years).

[26] Khoshdel A, Jafari SMS, Heydari ST, Abtahi F, Ardekani A, Lak FJ. The prevalence of cardiovascular disease risk factors, and metabolic syndrome among iranian military parachutists. Int Cardiovasc Res J. 2012;6(2):51–55.

[27] Maleki R, Mostafazadeh M, Nazari Sharif H, Rahim Nejad S, Gorgani-Firuzjaee S (2016). The prevalence of metabolic syndrome in air guard forces of Iran Army. Paramed Sci Military Health.

[28] Marzabadi A, Gholami Fesharaki M (2011). Effective factors on job stress in military personnel. Journal Mil Med.

[29] Payab M, Hasani-Ranjbar S, Merati Y, Esteghamati A, Qorbani M, Hematabadi M, Rashidian H, Shirzad N (2017). The prevalence of metabolic syndrome and different obesity phenotype in Iranian male military personnel. Am J Mens Health.

[30] Pourtaghi G, Valipour F, Sadeghialavi H, Lahmi M (2014). Anthropometric characteristics of Iranian military personnel and their changes over recent years. Int J Occup Environ Med.

[31] Zarchi AK, Gahangiri M (2010). Pre hypertension and hypertension in Iranian military personnel: prevalence according to some related factors. World Appl Sci J.

[32] Moher D, Liberati A, Tetzlaff J, Altman DG, Group P (2009). Preferred reporting items for systematic reviews and meta-analyses: the PRISMA statement. PLoS Med.

[33] Higgins JP, Thompson SG, Deeks JJ, Altman DG (2003). Measuring inconsistency in meta-analyses. BMJ: British Medical Journal.

[34] Higgins JP, Green S. Cochrane handbook for systematic reviews of interventions, vol. 4: John Wiley & Sons; 2011.

[35] Begg CB, Mazumdar M. Operating characteristics of a rank correlation test for publication bias. Biometrics. 1994:1088–101. 7786990

[36] Egger M, Smith GD, Schneider M, Minder C (1997). Bias in meta-analysis detected by a simple, graphical test. Bmj.

[37] DerSimonian R, Laird N (1986). Meta-analysis in clinical trials. Control Clin Trials.

[38] Murni I, Naing L, Halim MH: Prevalence of obesity and its association with hypertension: A cross sectional study of military personnel in the Royal Brunei Armed Forces.

[39] Ogunbiyi ETAOA, Hussain IBANA (2011). The prevalence of obesity in a Nigerian military population. TAF Preventive Medicine Bulletin.

[40] Hsu LL, Nevin RL, Tobler SK, Rubertone MV (2007). Trends in overweight and obesity among 18-year-old applicants to the United States military, 1993–2006. J Adolesc Health.

[41] Hruby A, Hill OT, Bulathsinhala L, McKinnon CJ, Montain SJ, Young AJ, Smith TJ (2015). Trends in overweight and obesity in soldiers entering the US Army, 1989-2012. Obesity.

[42] Stevens GA, Singh GM, Lu Y, Danaei G, Lin JK, Finucane MM, Bahalim AN, McIntire RK, Gutierrez HR, Cowan M (2012). National, regional, and global trends in adult overweight and obesity prevalences. Popul Health Metrics.

[43] Rappole C, Grier T, Anderson MK, Hauschild V, Jones BH (2017). Associations of age, aerobic fitness, and body mass index with injury in an operational Army brigade. J Sci Med Sport.

[44] Simon GE, Von Korff M, Saunders K, Miglioretti DL, Crane PK, Van Belle G, Kessler RC (2006). Association between obesity and psychiatric disorders in the US adult population. Arch Gen Psychiatry.

[45] Luppino FS, de Wit LM, Bouvy PF, Stijnen T, Cuijpers P, Penninx BW, Zitman FG (2010). Overweight, obesity, and depression: a systematic review and meta-analysis of longitudinal studies. Arch Gen Psychiatry.

[46] Wardle J, Steptoe A, Oliver G, Lipsey Z (2000). Stress, dietary restraint and food intake. J Psychosom Res.

[47] Huang C-J, Acevedo EO (2011). Occupational stress:the influence of obesity and physical activity/fitness on immune function. Am J Lifestyle Med.

[48] Newman E, O’Connor DB, Conner M (2007). Daily hassles and eating behaviour: the role of cortisol reactivity status. Psychoneuroendocrinology.

[49] Epel E, Lapidus R, McEwen B, Brownell K (2001). Stress may add bite to appetite in women: a laboratory study of stress-induced cortisol and eating behavior. Psychoneuroendocrinology.

[50] Block JP, He Y, Zaslavsky AM, Ding L, Ayanian JZ (2009). Psychosocial stress and change in weight among US adults. Am J Epidemiol.

[51] Hargens TA, Kaleth AS, Edwards ES, Butner KL (2013). Association between sleep disorders, obesity, and exercise: a review. Nature and Science of Sleep.

[52] Patel SR, HF B (2008). Short sleep duration and weight gain: a systematic review. Obesity.

[53] Shier V, Nicosia N, Datar A (2016). Neighborhood and home food environment and Children’s diet and obesity: evidence from military Personnel’s installation assignment. Soc Sci Med (1982).

[54] Hooks A (2015). Overweight in the military: causes and effects.

[55] Mahadevan S, Ali I (2016). Is body mass index a good indicator of obesity?. Int J Diabetes Dev Countries.

[56] Poston WSC, Foreyt JP (2002). Body mass index: uses and limitations. Strength Cond J.

[57] Heinrich KM, Jitnarin N, Suminski RR, Berkel L, Hunter CM, Alvarez L, Brundige AR, Peterson AL, Foreyt JP, Haddock CK (2008). Obesity classification in military personnel: a comparison of body fat, waist circumference, and body mass index measurements. Mil Med.

[58] Haddock CK, Poston WS, Klesges RC, Talcott W (1999). An examination of body weight standards and the association between weight and health behaviors in the United States air Force. Mil Med.

[59] IntHout J, Ioannidis JP, Borm GF, Goeman JJ (2015). Small studies are more heterogeneous than large ones: a meta-meta-analysis. J Clin Epidemiol.

[60] Zhang Z, Xu X, Ni H: Small studies may overestimate the effect sizes in critical care meta-analyses: a meta-epidemiological study. Crit Care 2013, 17(1):R2-R2. 10.1186/cc11919 PMC4056100 23302257

